# Improving the quality properties of soybean oil by using rice bran oil

**DOI:** 10.1038/s41598-024-53059-3

**Published:** 2024-02-01

**Authors:** Ahmed Sabry Mohammed, Hanafy Abdel Aziz Hashem, Badr Saed Abdel Maksoud

**Affiliations:** Food Science and Technology Department, Faculty of Agriculture, AL-Azhar University, Cairo, Egypt

**Keywords:** Analytical chemistry, Materials chemistry, Physical chemistry

## Abstract

This study aims to study the effect of substituting soybean oil (SO) with rice bran oil (RBO) at different levels (25%, 50%, and 75%) on the physical and chemical properties, fatty acid composition, and oxidative stability of SO, also, study the effect of storing SO, RBO, and their blend oils at ambient temperature for a period of 12 months on the content of free fatty acids (% FFA), peroxide value (PV), and thiobarbituric acid (TBA). RBO demonstrated good quality, as evidenced by its initial low values of % FFA, PV, and TBA. Furthermore, RBO was found to be an excellent source of γ-oryzanol, whereas the other oils lacked this compound. Consequently, increasing the proportion of RBO in SO resulted in the least degradation, while pure SO exhibited the highest degree of degradation. Moreover, the blend oils demonstrated an inhibitory effect against oxidation, allowing for a prolonged storage period without the use of industrial antioxidants. Throughout the entire storage period, the % FFA and PV of all tested blend oil samples remained within the limits recommended for human consumption. TBA exhibited a similar trend to PV. However, an incremental increase in TBA values was observed as the storage period of the oils extended. In SO, TBA levels increased from 0.533 mg malonaldehyde/kg oil at the beginning to 1.446 mg malonaldehyde/kg oil after 12 months of storage. In RBO, TBA levels increased from 0.336 mg malonaldehyde/kg oil at the beginning to 0.882 mg malonaldehyde/kg oil after 12 months of storage.

## Introduction

Rice (*Oryza sativa* L.) holds significant prominence as a major field crop in Egypt, occupying approximately 0.65 million hectares and yielding around 6 million metric tons of rough rice annually. This substantial production accounts for roughly 20% of per capita cereal consumption^1^. Rice is a vital cereal crop for nearly half of the global population, and numerous studies have highlighted its rich content of phytochemicals known for their potent antioxidant activity^2^. The rice plant consists of various components, including bran, grain, germ, and husk. Rice bran, in particular, contains a notable quantity of rice bran oil (RBO), constituting approximately 12–23% of its composition and harboring a substantial concentration of active compounds^3^.

RBO not only enhances the taste and flavor of food products but also exhibits reduced oil absorption during frying. Consequently, RBO has found applications as an ingredient in the cosmetic industry^4^. Over the past few years, it has been recognized as a functional oil due to its natural antioxidants and beneficial micronutrients^5^. One of the prominent constituents of RBO is γ-oryzanol, which is extracted from the inner husk and seeds of rice. γ-oryzanol represents a mixture of natural antioxidant compounds within RBO^6^.

Numerous studies have reported the health benefits of γ-oryzanol, including its potential to reduce blood lipid levels and enhance antioxidant capacity both in vivo and in vitro^7^. This study strongly recommends the incorporation of RBO, serving as a source of γ-oryzanol, into various food products to enhance their oxidative stability, nutritional value, and health benefits.

γ-oryzanol, present in RBO at levels of approximately 1–2%, exhibits significant potential for application in pharmaceuticals, nutraceuticals, and cosmeceuticals. It acts as a natural antioxidant, contributing to the wide range of applications and consumer acceptance of RBO in countries such as China, Taiwan, Japan, Korea, Thailand, and Pakistan. RBO is generally acknowledged as a high-quality vegetable oil, characterized by favorable cooking attributes, extended shelf life, desirable fatty acid composition, and exceptional stability at elevated temperatures^4,8^.

Therefore, the objective of this investigation was aims to the impact of substituting soybean oil (SO) with rice bran oil (RBO) at different levels (25%, 50%, and 75%) on quality properties (including physical and chemical properties, fatty acid composition, and stability) of SO. Additionally, this study aimed to evaluate the impact of storing the oils and their blends at ambient temperature for a duration of 12 months on % FFA, PV, and TBA levels. These chemical parameters serve as crucial indicators for assessing the quality and shelf-life stability of oils.

The novelty of this study aims to the utilization of rice bran oil as a replacement for soybean oil in the formulation of edible oil blends, aiming to enhance resistance against oxidation and rancidity during both utilization and storage. Moreover, the blend oils demonstrated an inhibitory effect against oxidation, allowing for a prolonged storage period without the use of industrial antioxidants.

## Materials and methods

### Materials

#### Rice bran oil (RBO)

Refined bleached and deodorized RBO used in this study were obtained in 2018 from Al-Bustan Company for investment and commercial development 7th Zahran Abdullah St., Izbat Al-Nakhl, Cairo, Egypt.

#### Soybean oils (SO)

Refined bleached and deodorized soybean oils used in this study were obtained in 2018 from Arma Food Industries, 10th of Ramadan City, Egypt.

#### Chemicals; solvents and reagents

All chemicals, solvents, and reagents used in this study for analytical grade were purchased from El-Gamhouria Trading Chemicals and Drugs Co, Egypt.

#### Containers

Polypropylene containers with a capacity of 50 ml were obtained from Inpaco. Company, 10th of Ramadan City, Egypt.

### Methods

#### Preparation of oil blends

**Figure 1:**
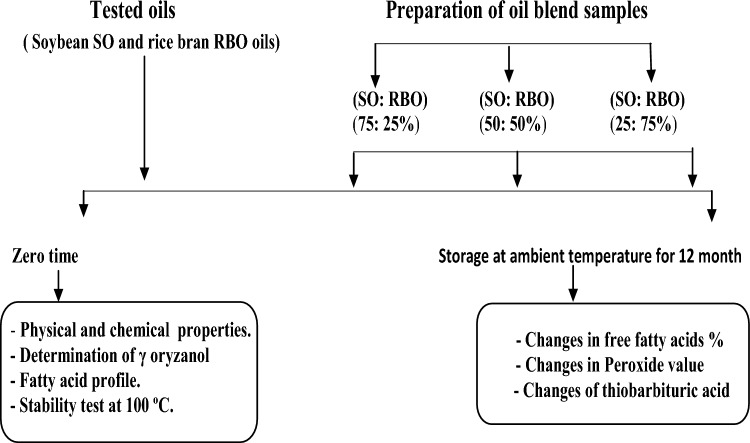
Schematic diagram for oil blends formulation and analysis.

According to^9^ with some modification, the soybean oil (SO) was blended with rice bran oil (RBO) in varying proportions. The SO: RBO (w/w) blends were prepared as follows: 75: 25, 50: 50, and 25: 75. The mixtures were stirred in a magnetic stirrer for 20 min for homogenization (Fig. 1).

#### Physical properties of tested oils and oil blend samples

Refractive Index According to^10^ was measured by using a Zeiss refractometer for SO and RBO and their blends at 25 °C. The specific gravity of SO and RBO oils as well as their blends was determined by using a glass pycnometer (5 ml) at 25 °C according to^10^. The melting point was determined using thin wall capillary tubes (1 mm internal diameter) according to the method described by^11^.

#### Chemical properties of tested oils and oil blend samples

##### Acidity% (free fatty acids FFA %)

The method used was adapted from^11^. FFA% (as oleic acid) of the pure vegetable oils and oil blends was determined. Before titration against 0.1 (N) NaOH, the tested sample was dissolved in neutralized ethanol-diethyl ether solvent (1:1 v/v).

##### Peroxide value (PV)

The PV of the tested samples was determined according to the method described in^10^. The liberated iodine was titrated with 0.01 (N) sodium thiosulfate solution using starch solution (1%) as an indicator. PV was expressed as milliequivalents (meq) peroxide oxygen per kg of oil.

*Iodine value (IV)* was calculated from the fatty acids composition of tested oils according to^12^.

##### Thiobarbituric acid value (TBA)

The method of^11^ was followed to determine the TBA value as of vegetable oils and oil blends samples. The absorbance of the developed color was measured at 532 nm against a blank reagent. TBA value was calculated and expressed as mg malonaldehyde/kg oil.

*Unsaponifiable matter (%)* was determined according to the method described in^10^. A known weight of the tested samples (ca.5g) was dissolved in 30 ml ethanol and then KOH solution (1.5 ml, 3:2, and w/v) was added. The tested samples were saponified in a water bath for 30 min under a reflux air condenser. The alcoholic soap solution was quantitatively transferred into a separatory funnel using 50 ml of water and 50 ml of petroleum ether. The Unsaponifiable matter was extracted with petroleum ether (3 × 20 ml) washed several times with distilled water then dried over anhydrous sodium sulfate and filtered into a weighed flask. The solvent was evaporated using a boiling water bath and dried at 105 °C until constant weight was reached.

#### Determination of gamma oryzanol (γ-oryzanol) content

Γ-oryzanol was determined by the spectrophotometric method according to the method described in^13^. This method is used to determine γ-oryzanol content (%) in oils from spectrophotometer absorption measurements at the wavelength of maximum absorption near 314 nm in a 1-cm quartz cuvette. Scope: applicable to RBO.

##### Apparatus

Spectrophotometer for measuring extinction in the ultraviolet between 310 and 320 nm.

- Rectangular quartz cuvettes—having an optical light path of 1 cm.
- Volumetric flask—100 ml.
- Filter paper—Whatman no. 2, or equivalent.

##### Reagents

n-Heptane—spectrophotometrically pure.

##### Procedure

Before use, the spectrophotometer should be properly adjusted to a zero-reading filling both the sample cuvette and the reference cuvette with n-heptane.

- Filter the oil sample through filter paper at ambient temperature.
- Weigh accurately approximately 0.2 g of the sample so prepared into a 100 mL volumetric flask, makeup to the mark with n-Heptane.
- Fill a cuvette with the solution obtained and measure the extinction at the wavelength of maximum absorption near 314 nm, using the same solvent as a reference.
- The extinction values recorded must lie within the range of 0.3–0.6. If not, the measurements must be repeated using more concentrated or more diluted solutions as appropriate. Calculate γ-oryzanol content as follows:
  $${\text{Gamma-oryzanol content }}\left( \% \right) \, = { 1}00 \, \times \, \left( {\text{1/W}} \right) \, \times {\text{ A }} \times \, \left( {\text{1/E}} \right)$$
  Where W = mass of sample (g), A = extinction (absorbance) of the solution, E = specific extinction E1%1 cm = 359.

##### Determination of fatty acids profiles

The fatty acids of investigated oils were determined as methyl ester by gas–liquid chromatography. The methyl ester samples were prepared using boron trifluoride (BF3) in methanol (20%) as a methylating agent according to the^10^.

##### Oxidative stability (Rancimat induction period)

The oxidative stability of tested oils, and oil blends samples was determined using an automated Rancimat (Metrohm Ud. CH-9100 Herisau, Switzeland, model 679) according to^14^. In this method, the tested sample is exposed to a stream of atmospheric oxygen (20 L/h) at 100 ± 0.2 °C. The induction time is the time needed to reach the breakpoint of this curve (point of greatest curvature).

*Storage experiment of oils and their blend samples* were subjected to storage experiment at ambient room temperature for 12 month. Every month during storage, oils and their blend samples of each treatment were withdrawn and subjected to changes in free fatty acids %, changes in peroxide value, and changes of thiobarbituric acid (Fig. 1).

### Statistical analysis

One-way a nova analysis of variance using Excel (office 2010) was performed on all experimental results sets. Post-hoc multiple comparisons were carried out by Duncan analysis to determine significant differences between sample means at the 5% level.

## Results and discussions

### Physical and chemical properties of tested oils and their blends

The physical and chemical properties of oils were among the most important properties that indicate the freshness and quality of the oils as well as their functionality in food products^15^. The physicochemical characteristics of edible oils play an important role in assessing their quality assurance, palatability, and consumer acceptability, as well as they were related to the healthy safe quality criteria of these lipids and foodstuffs processed by using them^16^.

The Physical and chemical properties analysis of SO, RBO, and their blends were presented in Table 1, the physical properties of tested oil samples were Refractive index (RI), specific gravity, melting point, and chemical properties were % free fatty acid (FFA); saponification value (SV); iodin value (IV); peroxide value (PV) and thiobarbituric acid (TBA).

**Table 1:** Physical and chemical properties of tested oils and their blends.

Quality parameter	Tested oils		Oil blends (SO: RBO)		
	SO	RBO	B1	B2	B3
Refractive index	1.4713 ± 0.07^a^	1.4672 ± 0.06^b^	1.4713 ± 0.07^a^	1.4672 ± 0.06^b^	1.4631 ± 0.05^c^
Specific gravity 25 °C	0.9144 ± 0.06^a^	0.9129 ± 0.05^d^	0.9142 ± 0.06^a^	0.9134 ± 0.05^c^	0.9137 ± 0.05^b^
Melting point (°C)	− 15.00 ± 0.10^a^	− 14.00 ± 0.08^b^	− 14.00 ± 0.08^a^	− 14.00 ± 0.08^a^	− 15.00 ± 0.10^b^
Free fatty acid (as oleic acid %)	0.040 ± 0.05^e^	0.180 ± 0.08^a^	0.080 ± 0.05^d^	0.110 ± 0.06^d^	0.120 ± 0.07^b^
Peroxide value (meqO_2_/kg oil)	0.960 ± 0.06^d^	1.20 ± 0.07^b^	1.46 ± 0.08^a^	1.17 ± 0.07^c^	0.54 ± 0.04^e^
Tiobarbeturic acid value (mg malonaldehyde/kg oil)	0.533 ± 0.10^a^	0.316 ± 0.07^c^	0.333 ± 0.08^b^	0.303 ± 0.07^d^	0.276 ± 0.06^e^
Iodine value (I_2_/100 g)	125.90 ± 0.18^a^	98.90 ± 0.11^d^	120.27 ± 0.16^b^	111.35 ± 0.14^b^	103.63 ± 0.12^c^
Saponification value (mg KOH/g fat)	202.32 ± 0.06^a^	202.00 ± 0.05^a^	ND	ND	ND
Unsaponifiable matter (%)	0.86 ± 0.07^a^	2.10 ± 0.13^b^	ND	ND	ND
Gamma oryzanol (mg/100 g oil)	0	500	125	250	375

Where: (M ± S.D) = mean ± std. deviation. Values with different small letters^a, b, c, d, e^ in the same row are significantly different (p < 0.05).

SO: soybean oil; RBO: rice bran oil; B1: (25:75%) (RBO: SO); B2: (50:50%); B3: (75:25%); ND: not determined.

As shown in Table 1 the RI at 25 ± 1 °C of SO and RBO were 1.4631, 1.4713, and 1.4672 respectively, while their oil blends were 1.4713, 1.4672, 1.4631 of B1 (25:75% RBO: SO), B2 (50:50%) and B3 (75:25) respectively, this is due to RI was used basically for estimation the degree of unsaturation, as well as it’s correlation with IV. These results agreed with those reported by^17^; both IV and RI were important characteristics that determine the degree of saturation or unsaturation of oils. The RI was used by most processors to measure the change in unsaturation as the oil was hydrogenated.

Also, results in Table 1, indicate the highest value of IV of SO was (125.90 I_2_/100 g) compared to the lowest value of RBO (98.90 I_2_/100 g).

The increased value of the melting point of RBO (− 14.0) compared to SO (− 15 °C) may be attributed to that RBO contains a higher amount of saturated fatty acid 22.85% compared to 15.74% for SO Table 2 these results were approximately similar to those obtained result by^18^. Also, the same finding was noted for melting point value (− 14 °C) for B1, B2, and (− 15 °C) for B3, which may be owing to the melting temperature of oils was directly related to the fatty acids, which decreases corresponding to unsaturation in addition to the number of PUFAs affects IV of vegetable oils^19^.

**Table 2:** Relative percentage of fatty acid profiles of tested oils and their blends.

Fatty acids	Tested oils		Oil blends (SO: RBO)		
	SO	RBO	B1	B2	B3
Myristic acid (C14:0)	ND	0.44 ± 0.04^d^	0.13 ± 0.02^a^	0.26 ± 0.02^b^	0.33 ± 0.03^c^
Palmitic acid (C16:0)	10.46 ± 0.06^a^	19.17 ± 0.07^e^	13.03 ± 0.07^b^	15.55 ± 0.08^c^	17.75 ± 0.08^d^
Palmitoleic acid (C16:1)	0.13 ± 0.02^c^	0.23 ± 0.03^d^	0.10 ± 0.02^b^	0.05 ± 0.01^a^	0.07 ± 0.01^a^
Margaric acid (C_17:0_)	0.84 ± 0.05^d^	ND	0.42 ± 0.04^c^	0.30 ± 0.03^b^	0.20 ± 0.02^a^
Stearic acid (C18:0)	3.56 ± 0.09^e^	2.11 ± 0.03^a^	3.02 ± 0.07^d^	2.75 ± 0.06^c^	2.42 ± 0.05^b^
Oleic acid (C18:1)	28.13 ± 0.05^a^	41.19 ± 0.07^e^	30.54 ± 0.04^b^	34.16 ± 0.05^c^	37.86 ± 0.04^d^
Linoleic acid (C18:2)	50.62 ± 0.09^e^	33.34 ± 0.04^a^	47.62 ± 0.07^d^	42.62 ± 0.04^c^	37.79 ± 0.05^b^
Linolenic acid (C18:3)	5.11 ± 0.05^e^	1.92 ± 0.02^a^	4.03 ± 0.04^d^	3.11 ± 0.03^c^	2.14 ± 0.02^b^
Arachidic acid (C20:0)	0.39 ± 0.02^a^	0.86 ± 0.06^e^	0.43 ± 0.04^a^	0.53 ± 0.05^b^	0.76 ± 0.06^c^
Gadoleic acid (C20:1)	0.27 ± 0.02^a^	0.47 ± 0.04^d^	0.26 ± 0.02^a^	0.32 ± 0.03^b^	0.41 ± 0.03^c^
Behenic acid (C22:0)	0.49 ± 0.04^d^	0.27 ± 0.02^a^	0.42 ± 0.04^c^	0.35 ± 0.03^b^	0.34 ± 0.02^b^
TSFAs	15.74 ± 0.03^a^	22.85 ± 0.08^e^	17.45 ± 0.04^b^	19.74 ± 0.05^c^	21.80 ± 0.07^d^
MUFAs	28.53 ± 0.04^a^	41.93 ± 0.07^e^	30.90 ± 0.05^b^	34.53 ± 0.04^c^	38.28 ± 0.05^d^
PUFAs	55.73 ± 0.08^e^	35.22 ± 0.04^a^	51.65 ± 0.06^d^	45.73 ± 0.05^c^	39.92 ± 0.04^b^

Where: (M ± S.D) = mean ± std. deviation.

Values with different small letters^a, b, c, d, e^ in the same row are significantly different (p < 0.05).

TSFAs: total saturated fatty acids; SO: soybean oil; RBO: rice bran oil; MUFAs: monounsaturated fatty acids; PUFAs: polyunsaturated fatty acids; B1: (25:75) % (RBO: SO); B2: (50:50%); B3: (75:25%); ND: not detected.

SV of SO and RBO was the same value approximately (202.32 and 202.00 mg KOH/g oil), respectively. The present result was approximately similar to those obtained by^20^.

In general, overall, the results presented in Table 1 indicate that the fresh oils used in the study were of good quality. This is evident from their low initial values of % FFA (Free Fatty Acid), PV (Peroxide Value), and TBA (Thiobarbituric Acid). These quality properties of the fresh oil samples align with the findings reported in reference^21^.

One notable characteristic of Rice Bran Oil (RBO), as revealed by the results in Table 1, is its high content of γ-oryzanol compared to the other oils. The other oils included in the study did not contain γ-oryzanol.

Additionally, the blends containing RBO exhibited a higher % FFA compared to Soybean Oil (SO), which can be attributed to the initial % FFA content of RBO.

The melting point of RBO was found to be higher than that of SO, as indicated by the results in Table 1. This difference can be attributed to the higher amount of saturated fatty acids present in RBO (22.85%) compared to SO (15.74%). Similar findings have been reported in references^16,18^.

The melting point values of the blends (B1, B2, and B3) were approximately – 14 °C and − 15 °C, respectively, which can be attributed to the melting temperature of the oils being directly related to the fatty acids they contain. The melting temperature decreases with unsaturation, and the number of polyunsaturated fatty acids (PUFAs) affects the Iodine Value (IV) of vegetable oils. This finding is supported by reference^19^ and confirmed by reference^22^.

The variation in PV values among the samples can be attributed to differences in the triglyceride structure, which depend on the oil sources and the variation in the proportion of unsaturated bonds in the fatty acids of the triglycerides. Unsaturated bonds are more prone to oxidation, leading to higher PV values.

Regarding the blends presented in Table 1, it can be observed that the IV decreases with increased levels of RBO. This can be attributed to the decreased content of linoleic and linolenic acids in RBO compared to SO, as indicated in Table 2.

In summary, the results in Table 1 provide insights into the quality characteristics of the oils studied, including their % FFA, PV, TBA, γ-oryzanol content, melting point, and IV. These findings are consistent with previous studies cited in references^16,18,19,21,22^, highlighting the importance of fatty acid composition and triglyceride structure in determining the properties of vegetable oils.

### Fatty acid profiles of tested oils and their blends

Fatty acid plays multiple roles in the human body and other organisms. In addition to proteins and carbohydrates, FA constitutes the main components of biological matter. It had been found that the regular intake of saturated fatty acid increases of the level cholesterol, which was linked with increased coronary heart disease mortality^23^.

The fatty acid profiles of the two pure vegetable oil samples used in formulating oil blends SO and RBO were determined. The tabulated data reveals that there were remarkable differences in the fatty acid profiles of the studied oils. Only ten fatty acids (5 SFAs and 5 USFAs) were detected in SO and RBO were detected. So, oil had the highest TUSFA content (84.26% of total fatty acid) comparable amounts by RBO (64.15%), Table 2. In addition, the two fatty acids (C_16:0_ and C_18:0_) were the highest SFAs in SO and RBO reaching 10.46, 3.56, and 19.17, 2.11% in SO and RBO, respectively.

For unsaturated fatty acids (USFAs), the highest values were recorded for C_18:1_ (41.19%) in RBO, and the lowest value was recorded in SO (28.13%), while the highest values were recorded for C_18:2_ (50.62%) in SO while RBO was recorded (33.34%).

The results in Table 2, indicate that the main components of polyunsaturated fatty acid (PUFAs) have differed as a result of the process of blending oil samples. We find that the oleic acid (C_18:1_) increases with the increase in the percentage of RBO where it was 30.54, 34.16 and 37,86% for B1, B2, and B3 respectively, while on the contrary, the percentage of linoleic acid (C_18:2_) and linolenic acid (C_18:3_) ((ω-3) was decreased with an increase RBO content it was 47.62–4.03, 42.62–3.11 and 37.79–2.14% for B1, B2, and B3 respectively.

In general, from the results obtained in Table 2 we find that with an increase in the percentage of RBO, the resulting blends of oil are closer to the recommendations of^24^.

### Oxidative stability of tested oils and their blends

Oxidative stability (also known as the induction period) was a measurement of oil resistance to oxidation. Because the process takes place through a chain reaction, the oxidation reaction has a period when it’s relatively slow before it suddenly speeds up. Oxidative stability is one of the most important indicators for maintaining the quality of edible oils^25^. In addition, the knowledge about the oxidative state of the edible oils provides an idea for the expectation of their shelf-life and susceptibility to oxidative rancidity during storage periods and processing as well as for their possible uses for edible or industrial purposes^26^. The induction period measurements are carried out on the fresh oils and blends to provide a quick induction of the trends in resistance to oxidative rancidity as well as the shelf-life of oils.

The induction period value of tested oils and their blends used in the investigation was measured and the obtained results were recorded in Table 3.

**Table 3:** Oxidative stability at 100 ˚C of tested oils and their blends.

Oxidative stability	Tested oils		Oil blends (SO : RBO)		
	SO	RBO	B1	B2	B3
Induction period in hrs	18.30 ± 0.05^c^	24.08 ± 0.08^a^	18.78 ± 0.06^c^	20.88 ± 0.06^b^	23.44 ± 0.07^a^
validity period in month	9.76 ± 0.07^d^	12.84 ± 0.11^a^	10.02 ± 0.08^c^	11.13 ± 0.09^b^	12.50 ± 0.10^a^

Where: (M ± S.D) = mean ± std. deviation. Values with different small letters^a, b, c, d, e^ in the same row are significantly different (p < 0.05).

SO: soybean oil; RBO: rice bran oil; B1: (25:75) % (RBO:SO); B2: (50:50%); B3: (75:25%).

From these results, RBO showed the highest stability among the tested oils. Its induction period value (IP) in hours reached 24.08 and its validity period (VP) was 12.84 month, followed by SO. It can be noticed from these results that showed an agreement with^27^. Also, in the same Table 3 noticed that the highest IP hr. and VP month of blend oil samples were recorded for B3 (23.44 h. and 12.50 month) followed by B2 recorded (20.88 h. and 11.13 month) and then B1 (18.78 h. and 10.02 month). On the other hand, RBO had a value of IP and VP (24.08 h. 12.84 month). While SO had the lowest value (IP 18.3 h. and VP 9.76 month). This may be attributed to the unsaturation degree of the oil samples in this study.

In general, the result of Table 3 noted that the highest IP hr. and VP month of blend oil samples were recorded for B3 followed by B2 and then B1, while RBO had a value of IP and VP. On the other hand, the SO had the lowest value; this may be attributed to the unsaturation degree of the oils under study.

### Changes in chemical properties of tested oils and their blends during storage at ambient temperature

The effect of the storage period at ambient temperature on some chemical properties of oil blends under investigation was studied. The storage period at ambient temperature experiment was extended for 12 months (the shelf-life of oil blends as recommended by^21^. Every month during the storage period, a sample representing each treatment was withdrawn and tested for its FFA, PV, and TBA (as chemical properties). The important chemical parameters to assess the quality and shelf-life stability of any oil are FFA, PV, and TBA values. Hence in the present study, these quality parameters were tested at the laboratory using standard procedures and presented in the next table, each parameter is depicted graphically.

#### Changes in free fatty acids (% FFA)

% FFA content of all edible oils and their blends increased significantly (*p* < 0.05) and steadily during storage. The amount of FFA increases as a result of the hydrolysis of triacylglycerols, which contributes to the development of off-flavors and off-odors in the oil^28^.

The data obtained during the storage period of oil samples and their blends revealed that there was an increase in % FFA with an increase in storage time. The increase was more or less marginal and was not very high enough to affect the quality of oil drastically. The change in %FFA of RBO and its blends with SO was presented in Table 4. The initial value of % FFA for SO, RBO, and their blends B1, B2, and B3 were 0.04, 0.18, 0.08, 0.11, and 0.12, respectively. The data obtained during the storage of oil samples and their blends revealed that there was an increase in % FFA with an increase in storage time. The increase was more or less marginal and was not very high enough to affect the quality of oil drastically.

**Table 4:** Changes in % FFA as oleic acid of tested oils and their blends during storage at ambient temperature for 12 months.

Storage period (month)	% FFA of SO, RBO, and their blends				
	SO	RBO	Oil blends (SO: RBO)		
			B1	B2	B3
0	0.04^d^ ± 0.02	0.18^a^ ± 0.01	0.08^c^ ± 0.00	0.11^b^ ± 0.00	0.12^b^ ± 0.00
1	0.04^d^ ± 0.01	0.18^a^ ± 0.00	0.10^c^ ± 0.00	0.12^b^ ± 0.00	0.13^b^ ± 0.01
2	0.05^e^ ± 0.01	0.19^a^ ± 0.01	0.11^d^ ± 0.00	0.13^c^ ± 0.01	0.15^b^ ± 0.01
3	0.06^d^ ± 0.01	0.19^a^ ± 0.00	0.12^c^ ± 0.00	0.13^c^ ± 0.00	0.15^b^ ± 0.00
4	0.06^e^ ± 0.00	0.20^a^ ± 0.00	0.12^d^ ± 0.00	0.15^c^ ± 0.00	0.16^b^ ± 0.00
5	0.08^d^ ± 0.00	0.21^a^ ± 0.00	0.13^c^ ± 0.00	0.16^b^ ± 0.00	0.17^b^ ± 0.00
6	0.08^d^ ± 0.00	0.21^a^ ± 0.00	0.14^c^ ± 0.00	0.16^b^ ± 0.00	0.17^b^ ± 0.00
7	0.09^d^ ± 0.00	0.21^a^ ± 0.00	0.15^c^ ± 0.01	0.17^b^ ± 0.00	0.18^b^ ± 0.00
8	0.12^e^ ± 0.01	0.22^a^ ± 0.00	0.15^d^ ± 0.01	0.18^c^ ± 0.00	0.19^b^ ± 0.00
9	0.15^c^ ± 0.01	0.22^a^ ± 0.01	0.17^b^ ± 0.00	0.18^b^ ± 0.00	0.21^a^ ± 0.01
10	0.15^d^ ± 0.01	0.23^a^ ± 0.01	0.18^c^ ± 0.01	0.20^b^ ± 0.01	0.23^a^ ± 0.01
11	0.18^c^ ± 0.01	0.23^a^ ± 0.01	0.20^b^ ± 0.01	0.21^b^ ± 0.00	0.24^a^ ± 0.01
12	0.18^c^ ± 0.00	0.24^b^ ± 0.00	0.23^b^ ± 0.00	0.23^b^ ± 0.01	0.26^a^ ± 0.00

Where: (M ± S.D) = mean ± std. deviation. Values with different small letters^a, b, c, d, e^ in the same row are significantly different (p < 0.05).

SO: soybean oil; RBO: rice bran oil; B1: (RBO:SO) (25:75) %; B2: (50:50%); B3: (75:25%).

The final %FFA values obtained for the samples up to 12-month storage were between 0.18 and 0.26%. However, partial replacement of SO with RBO could result in decreased FFA during storage, which indicates that the rate of generation of FFA was faster in pure SO compared to that in blend oils.

In general, based on the information provided, it can be inferred that Table 4 presents the results of an experiment involving the partial replacement of SO (presumably referring to soybean oil) with RBO (possibly referring to rice bran oil). The experiment aimed to evaluate the effect of this replacement on the formation of free fatty acids (FFA) and the degradation of polyunsaturated fatty acids (PUFA) during storage. The results indicate that the incorporation of RBO into SO led to a decrease in FFA values during storage. This suggests that the rate of FFA generation was higher in pure SO compared to the blend oils containing RBO. Therefore, the presence of RBO in the samples contributed to reducing the degradation of the oils, with the blend oils showing less degradation compared to pure SO.

Furthermore, the combination of SO and RBO resulted in a decrease in the level of PUFA, which are polyunsaturated fatty acids, and an increase in the level of MUFA, which are monounsaturated fatty acids. This implies that the blending process between RBO and SO led to a slower decrease in the relative content of PUFA compared to the oxidative degradation of PUFA that typically occurs during heating processes^9^.

#### Changes in peroxide value (PV)

For peroxides, the data confirmed the results obtained in early studies^9,29^ with an increase in the peroxides until a maximum was reached, followed by a decrease in those compounds due to their reactions and degradations to other compounds.

PV was used as a measure of the primary oxidation of oil, fat, and fatty food. The PV of the SO as influenced by the RBO as well as blends during storage for 12 months were shown in Table 5. There was an initial sharp increase in the PV from 0 to 10 months in SO, after which the rate slowed down.

**Table 5:** Changes in PV (meq. O_2_/kg oil) of tested oils and their blends during storage at ambient temperature for 12 months.

Storage period (month)	PV of SO, RBO, and their blends				
	SO	RBO	Oil blends (SO: RBO)		
			B1	B2	B3
0	0.96^c^ ± 0.06	1.20^b^ ± 0.02	1.46^a^ ± 0.15	1.17^b^ ± 0.02	0.54^d^ ± 0.04
1	1.16^bc^ ± 0.28	1.43^b^ ± 0.15	2.03^a^ ± 0.11	1.43^b^ ± 0.15	0.96^c^ ± 0.02
2	2.33^a^ ± 0.05	1.83^b^ ± 0.15	2.30^a^ ± 0.20	2.33^a^ ± 0.20	1.41^c^ ± 0.01
3	2.80^a^ ± 0.26	2.33^b^ ± 0.25	3.10^a^ ± 0.10	2.92^a^ ± 0.11	2.25^b^ ± 0.05
4	3.60^a^ ± 0.40	3.60^a^ ± 0.40	3.92^a^ ± 0.11	3.73^a^ ± 0.15	3.83^a^ ± 0.03
5	3.93^b^ ± 0.11	4.30^a^ ± 0.26	4.30^a^ ± 0.26	4.11^ab^ ± 0.02	4.25^ab^ ± 0.05
6	4.90^ab^ ± 0.17	4.43^b^ ± 0.23	5.46^a^ ± 0.75	4.70^b^ ± 0.26	5.06^ab^ ± 0.04
7	7.23^a^ ± 0.58	5.50^c^ ± 0.30	6.80^ab^ ± 0.20	6.06^bc^ ± 0.83	6.16^bc^ ± 0.15
8	9.16^a^ ± 0.64	6.28^c^ ± 0.15	7.66^b^ ± 0.30	7.53^b^ ± 0.25	7.38^b^ ± 0.36
9	9.90^a^ ± 0.10	7.33^d^ ± 0.35	8.93^b^ ± 0.30	8.70^bc^ ± 0.25	8.37^c^ ± 0.28
10	10.13^a^ ± 0.23	8.63^c^ ± 0.32	9.60^ab^ ± 0.40	9.53^ab^ ± 0.55	9.16^bc^ ± 0.06
11	9.43^a^ ± 0.20	9.58^a^ ± 0.21	10.08^a^ ± 0.35	10.15^a^ ± 0.30	9.76^a^ ± 0.66
12	8.23^c^ ± 0.25	10.04^b^ ± 0.14	9.93^b^ ± 0.03	11.60^a^ ± 0.36	10.36^b^ ± 0.55

Where: (M ± S.D) = mean ± std. deviation. Values with different small letters^ a, b, c, d, e^ in the same row are significantly different (p < 0.05).

SO: soybean oil; RBO: rice bran oil; B1: RBO:SO (25:75)%; B2: (50:50)%; B3: (75:25)%.

Soybean oil showed a faster rate of increase in PV compared to blends. This could be attributed to the high amounts of linoleic acids present in the SO compared to RBO^9^.

The addition of RBO to SO significantly (*p* < 0.05) slowed the increment of the concentration of hydroperoxides in SO. We find that the blend (B1) was the initial value (1.46), and then it reached its maximum in the month 11th, and after that, there was a gradual decrease until it reached (9.93 meq. O_2_/kg oil) at the end of the storage period, while B2 and B3 the initial value was 1.17 and 0.54 meq. O_2_/kg oil, respectively, and then that value reached 11.60 and 10.36 meq. O_2_/kg oil, respectively, at the end of the storage period, and there was no decrease in the PV**,** these results are in agreement with^30^. The nutritional contribution of minor components such as tocopherol, tocotrienols, and oryzanol in RBO blends may have conferred this greater oxidative stability^31^. Thus, the SO containing a higher amount of the RBO had a more inhibitory effect against oxidation due to the presence of minor components in RBO, as turned out from Table 5.

In general, the addition of RBO to SO significantly (*p* < 0.05) slowed the increment of the concentration of hydroperoxides in SO. A blending of RBO and SO improves the oxidative stability of SO and retard the rancidity in fried product during storage.

#### Changes of thiobarbituric acid (TBA)

The formation of secondary oxidation products under storage conditions was determined by TBA and is presented in Table 6. TBA value was used to assess the extent of secondary oxidation substances in oil and oily foods^32,33^. The degradation of hydroperoxides which produces secondary oxidation compounds as well as transformation of primary lipid oxidation products to secondary lipid oxidation substances during the storage of oil products leads to an increase in the level of secondary oxidation products^34^.

**Table 6:** Changes in TBA value (malonaldehyde/kg oil) of tested oils and their blends during storage at ambient temperature for 12 month.

Storage period (month)	TBA value of SO, RBO, and their blends				
	SO	RBO	Oil blends (SO: RBO)		
			B1	B2	B3
0	0.533^a^ ± 0.03	0.316^bc^ ± 0.03	0.333^b^ ± 0.02	0.303^bc^ ± 0.01	0.276^d^ ± 0.01
1	0.583^a^ ± 0.00	0.326^b^ ± 0.02	0.336^b^ ± 0.01	0.330^b^ ± 0.01	0.310^b^ ± 0.02
2	0.600^a^ ± 0.01	0.336^c^ ± 0.01	0.390^b^ ± 0.01	0.336^c^ ± 0.01	0.326^c^ ± 0.02
3	0.626^a^ ± 0.00	0.363^c^ ± 0.00	0.430^b^ ± 0.02	0.346^c^ ± 0.02	0.336^c^ ± 0.01
4	0.660^a^ ± 0.01	0.390^c^ ± 0.01	0.453^b^ ± 0.01	0.356^d^ ± 0.01	0.350^d^ ± 0.03
5	0.686^a^ ± 0.00	0.430^c^ ± 0.02	0.583^b^ ± 0.00	0.376^d^ ± 0.00	0.353^d^ ± 0.03
6	0.703^a^ ± 0.01	0.443^c^ ± 0.01	0.626^b^ ± 0.00	0.403^d^ ± 0.03	0.370^e^ ± 0.01
7	0.846^a^ ± 0.03	0.516^c^ ± 0.03	0.703^b^ ± 0.01	0.403^d^ ± 0.03	0.390^d^ ± 0.01
8	0.856^a^ ± 0.03	0.536^b^ ± 0.04	0.820^a^ ± 0.02	0.486^bc^ ± 0.08	0.440^c^ ± 0.02
9	0.913^a^ ± 0.01	0.600^b^ ± 0.02	0.833^a^ ± 0.01	0.503^c^ ± 0.10	0.496^c^ ± 0.01
10	0.983^a^ ± 0.02	0.670^c^ ± 0.02	0.880^b^ ± 0.02	0.516^d^ ± 0.03	0.536^d^ ± 0.01
11	1.203^a^ ± 0.10	0.746^c^ ± 0.02	0.910^b^ ± 0.01	0.770^c^ ± 0.12	0.680^c^ ± 0.05
12	1.446^a^ ± 0.18	0.883^b^ ± 0.07	0.976^b^ ± 0.01	0.870^b^ ± 0.05	0.850^b^ ± 0.04

Where: (M ± S.D) = mean ± std. deviation. Values with different small letters^a, b, c, d, e^ in the same row are significantly different (p < 0.05).

SO: soybean oil; RBO: rice bran oil; B1: RBO:SO (25:75) %; B2: (50:50) %; B3: (75:25) %.

The TBA value rose for all oil samples as the time of storage elapsed Table 6. However, the TBA levels were well below the rancidity onset which usually occurs at TBA levels of 1.00 and higher^33^**,** except SO until month, 9^th^ indicating the stability of oils and their blends during the periods studied.

According to the result in Table 6 TBA values differed according to the type of oil and its proportion in the oil blend, storage period, and interaction between these factors.

In general, the thiobarbituric acid (TBA) values exhibited a similar trend to the peroxide values (PV) presented in Table 5 of the study. Furthermore, a progressive elevation in TBA values was observed as the storage period of the oils extended. Specifically, in the case of soybean oil (SO), the TBA levels increased from an initial value of 0.533 mg malonaldehyde/kg oil at the beginning of the storage period to 1.446 mg malonaldehyde/kg oil after 12 months of storage. Similarly, for rice bran oil (RBO), the TBA levels increased from 0.336 mg malonaldehyde/kg oil at the initiation of storage to 0.882 mg malonaldehyde/kg oil after 12 months of storage.

## Conclusions

In general, it can be inferred that the substitution of soybean oil with rice bran oil in the formulation of edible oil blends confers a greater inhibitory effect against oxidation, thereby enabling an extended storage period without the incorporation of industrial antioxidants. The percentages of free fatty acids (% FFA) and peroxide values (PV) in all tested edible oil blend samples containing rice bran oil remained within the acceptable limits for human consumption, as recommended by^21^. However, a gradual increase in thiobarbituric acid (TBA) values was observed as the storage period of the oils extended.

## Data Availability

The data and materials were mentioned in the manuscript and the data was available upon request from the corresponding author.

## Abbreviations

SO: Soybean oil
RBO: Rice bran oil
B1: Blend1 (25:75%) (RBO: SO)
B2: Blend2 (50:50%) (RBO: SO)
B3: Blend3 (75:25%) (RBO: SO)
% FFA: Free fatty acids content
PV: Peroxide value
TBA: Thiobarbituric acid
RI: Refractive index
SV: Saponification value
IV: Iodine value
ND: Not determined
TSFAs: Total saturated fatty acids
MUFAs: Monounsaturated fatty acids
PUFAs: Poly unsaturated fatty acids
